# Resource Allocation, Treatment, Disclosure, and Mitochondrial Replacement Techniques: Some Comments on de Melo-Martin and Harris

**DOI:** 10.1017/S0963180116000876

**Published:** 2017-04

**Authors:** CÉSAR PALACIOS-GONZÁLEZ

## Introduction

There has been a lively exchange in this journal between Inmaculada de Melo-Martin and John Harris on the ethics of mitochondrial replacement techniques (MRTs). Initially, Harris advocated, here and elsewhere, for MRTs.^1^ He tried to show that the arguments against them are flawed and that MRT research and clinical practice should be supported because MRTs diminish suffering and increase well-being.

In response, de Melo-Martin^2^ argued that Harris’s arguments defending MRTs are found wanting and that the scientific community should oppose them. She contended three things: that Harris only engaged with the *weakest* arguments that have been advanced against MRTs, that the resources that are used for MRT research and clinical practice should be repurposed for achieving worthier goals, and that MRTs are not necessary for women who have a mitochondrial DNA disease and want to have children genetically related to them, as they could have children through other means (e.g., adoption or egg donation).

Harris then replied^3^ to de Melo-Martin and defended his arguments. First, he argued that in most instances both of them maintain the same position regarding de Melo-Martin’s objections to MRTs, and that where they do diverge, it is de Melo-Martin who is on the wrong side. Second, Harris contends that de Melo-Martin’s main criticism is off target. He maintains that he was not making any claim about *what priority* we should give to MRT research and clinical practice, only that his sole aim was to assess if *in principle* MRTs, conceived solely as biotechnologies and abstracted from our social reality, are morally objectionable or not.

This article constitutes a fourth act in this interplay of opinion. Here I will broaden the scope of the debate by presenting a richer account of the MRTs phenomenon: when each of the techniques that have jointly been labelled as MRTs (i.e., pronuclear transfer [PNT] and maternal spindle transfer [MST]) are independently examined, it becomes clear that the ethical panorama is far more complex than it first appears. I will also develop areas that naturally follow from de Melo-Martin’s and Harris’s discussion of the topic.

This article proceeds as follows. In the first section, I describe what mitochondrial diseases and MRTs are. This is important because in order to explain the differences among MRTs I need to expand on Harris’s portrayal of “mitochondrial disease,” which de Melo-Martin seems to follow. In the second section I address how MRTs could *prevent* mitochondrial diseases and if they would be effective in doing so. I do this by unpacking the differences among MRTs. In the final section I present and defend that parents have strong reasons to disclose to their children that they were MRT conceived, and show how this relates to Harris’s and de Melo-Martin’s discussion of whether there is such thing as a “right to know our genetic origins.”

## Mitochondrial Diseases and MRTs

Mitochondria are cellular organelles that generate the energy that cells need to work properly. They are characterized by possessing their own DNA (mitochondrial DNA [mtDNA]), by only being inherited via the maternal line,^4^ and by the fact that their means of inheritance are non-Mendelian.^5^ Whereas nuclear DNA (nDNA) represents 99.9% of total human DNA, mtDNA only represents 0.1%.^6^

In both “Germline Modification and the Burden of Human Existence” and “Germline Manipulation and Our Future Worlds,” Harris states that “[m]itochondrial disease can be very serious, causing conditions like Leigh’s disease, a fatal infant encephalopathy, and others that waste muscles or cause diabetes and deafness.”^7^ De Melo-Martin, in “When the Milk of Human Kindness becomes a Luxury (and Untested) Good. A Reply to Harris’ Unconditional Embrace of Mitochondrial Replacement Techniques,” does not provide a characterization of mitochondrial diseases.

I will expand on Harris’s characterization of “mitochondrial disease,” because for the sake of this discussion it is important to be specific about the details of what mitochondrial diseases are and how MRTs could prevent them. In a broad sense, there are two classes of mitochondrial diseases: mitochondrial diseases that are caused by problems in the mtDNA, and mitochondrial diseases that are caused by problems in the nDNA.^8^ This distinction is important because MRTs *cannot* be employed to deal with mitochondrial diseases caused by problems in the nDNA. MRTs *can only* be employed when the problems are caused by the genes in the mitochondria themselves. In other words, MRTs can only be employed when dealing with mtDNA diseases.

MtDNA diseases are *a group* of neuromuscular diseases that cause suffering and premature death.^9^ MtDNA is not uniform: there exist various mutations among the mitochondria, some of which are deleterious (i.e., mutations that prevent mitochondria from producing adequate levels of energy). Mutations, both deleterious and non-deleterious, can be maternally inherited or created spontaneously. In some instances, deleterious mutant DNA can be the only type of mtDNA that mitochondria possess. This is referred to as “homoplasmy.” Additionally, deleterious mutant DNA can be present only in some mitochondria, known as “heteroplasmy.”

Women with homoplasmic mtDNA containing deleterious mutations will *always* pass on the deleterious mutant mtDNA to their genetic offspring, irrespective of whether the kind of mutation will cause medical problems or not. Women with heteroplasmic mtDNA mutations, on the other hand, will pass on a mixture of mitochondria to their offspring, some of them *without* deleterious mutations and some of them *with* deleterious mutations. In this case, the manifestation of the disease will depend on the deleterious mtDNA mutant load and the kind of mutation that is present.^10^

## MRTs^11^

In his two articles on the subject of MRTs^12^ and his response to de Melo-Martin,^13^ Harris does not characterize the techniques that could be employed to avoid mtDNA diseases.^14^ This was not necessary, as he was interested in discussing the ethics of germline modifications, and both types of MRT can cause such modifications when selecting for females. De Melo-Martin^15^ also does not present a characterization of these techniques. In fact, she mentions only MST when discussing the safety issues related to MRTs, referencing the research of Tachibana et al.^16^

In de Melo-Martin’s case, the absence of a proper characterization of both MST and PNT is relevant because the differences between them yield different philosophical conclusions when considering issues about harm and identity, as Wrigley et al.^17^ and Palacios-González^18^ have examined. I return to this point when I discuss how mtDNA diseases can be “prevented” through MRTs.

The two most recently developed techniques that could help women affected by mtDNA diseases to have disease-free, genetically related children are MST and PNT, as said previously. Here I present a summarized version of how these techniques work.

In PNT, an oocyte from a woman with an mtDNA disease (Woman A) and an oocyte from a donor that possesses healthy mitochondria (Woman B) undergo in vitro fertilization (IVF). The oocytes can be fertilized with sperm from Woman A’s partner or with sperm from a donor. After the sperm has fertilized the oocytes, and during the first hours, the nuclear material of both progenitors is enclosed in different membranes that are called the male and female pronuclei. On day one in the development phase, and prior to the fusion of the pronuclei, the pronuclei of both zygotes are removed. The pronuclei housed in the cell produced with the donor’s egg are discarded along with Woman A’s now enucleated cell (remember that this cell possesses deleteriously mutated mtDNA). The pronuclei from Woman A, and her partner’s or donor’s pronuclei, are ferried into the now enucleated cell that was produced with Woman B’s oocyte (remember that this cell possesses healthy mitochondria). The fused cell is transferred into the woman intending to be the genetic mother, or a surrogate, and if everything goes according to plan the embryo will develop normally.^19^

In MST, assisted reproductive techniques are used to obtain eggs from the woman with an mtDNA disease (Woman A) and from a donor with healthy mitochondria (Woman B). The nDNA (which is found on one side of the oocyte in a spindle-shaped group) from both oocytes is removed. The chromosomes of Woman A are then ferried into Woman B’s enucleated egg. Woman B’s chromosomes and Woman A’s enucleated oocyte (again remember that this cell possesses deleteriously mutated mtDNA) are discarded. The reconstructed egg, which has healthy mitochondria, goes through IVF and then is transferred into the intending mother, or a surrogate. The fused cell will go on to develop normally, if everything goes as planned.^20^

Two things bear mentioning. First, in both MST and PNT, it is possible that mitochondria with deleterious mutations could accidentally be transferred when the chromosomal carry-over is taking place. If this were the case, it is not impossible that the mtDNA disease could manifest in the child. Second, if MST and PNT are successful, then the healthy mitochondria provided by the egg donor will be passed down via the maternal line to all subsequent generations. This means that if females are selected for when MST or PNT are used, then the third-party mitochondria will be inherited when these women reproduce. If, on the other hand, males are selected for, then the mitochondria *will not* be passed down to the next generation.

### How Do MRTs Prevent mtDNA Diseases?

Harris states that “MRT will prevent serious mitochondrial disease and the suffering it causes for women with mitochondrial disease, for their own children, and for countless future generations.”^21^ I will now explore how MRTs could do this.

Whereas Harris is in general optimistic about the development of MRTs, de Melo-Martin is sceptical. She argues with him that the resources invested in the development of MRTs and their translation into clinical practice only benefits a very small number of people:

[I]f reduction of the burdens of mitochondrial disorders were indeed the goal, research on basic and clinical studies on the causes, prevention, and treatment of these diseases will in all likelihood be more effective than research on MRTs. Even if all the women who could be eligible to use them did so—a big “if” indeed—these technologies would still have a relatively limited application. On the other hand, research on the diseases themselves and on more effective treatments for mitochondrial disorders would be of use to all of those with these diseases.^22^

First, one needs to be aware that MST and PNT prevent mtDNA diseases in two different ways. *In most cases MST prevents mtDNA diseases by creating someone*^23^ *without the disease*. MST, in most cases, *does not cure* someone of mtDNA disease. If the person who comes into existence is tied to her nuclear genetic makeup (i.e., if our numerical identity depends on our nuclear genetic makeup) then MST cannot be said to cure anyone, in most cases, because the fact that a couple or a woman decide to use MST alters the timing of conception and thus alters which gametes will fuse. For example, it is utterly improbable that the *same sperm and egg* would have fused in the following scenarios: (1) a couple decided to use MST, or (2) the same couple decided to naturally reproduce, instead of employing MST. Furthermore, it is highly improbable that the *same sperm and egg* would have fused if the couple decided to use MST but chose to have the procedure *5 weeks* after MST took place in scenario (1).

I have added the qualification “in most cases” because there is the possibility that a single sperm and a single egg could have been chosen beforehand for the procedure.^24^ In this instance, it could be said that MST ‘cured’ someone because the being who would result from the fertilization if MST *did not take* place and the being who would result from the fertilization if *MST did take place* would possess the same nuclear DNA. This being the case, one has to conclude that in most instances MST prevents mtDNA diseases by creating someone without an mtDNA disease, and that *only when a single sperm and egg have been selected beforehand can it be said that MST prevents an mtDNA disease by curing someone*.

PNT *as a mere technique*, on the other hand, prevents mtDNA diseases by curing someone affected by them. If we accept, as before, that our numerical identity depends on our nuclear genetic makeup, then we have to accept that an embryo originated with X’s sperm’s nDNA, Y’s egg’s nDNA, and the faulty mtDNA W (found in Y’s egg) is one and the same embryo as that originated with X’s sperm’s nDNA, Y’s egg’s nDNA, and *the healthy mtDNA Z* (found in the donor’s egg). In this case *it can be affirmed that PNT prevents mtDNA diseases by curing someone*. Wrigley et al. have defended this point: “In particular, PNT is a treatment which is attempting ‘pre-emptively’ to cure a person without affecting his or her identity. Thus, PNT is like, or is even a form of, gene therapy. MST, on the other hand, is a form of selective reproduction and has more in common with pre-implantation genetic diagnosis and pre-natal screening than it does with gene therapy.”^25^

Although this is the case,^26^ when one considers conducting PNT in an embryo that was *specifically* produced by a woman, or couple, in order to have a child without an mtDNA disease (i.e., *the clinical practice* of PNT), if it were not for the fact that PNT was going to occur, most probably that embryo would not have existed. The timing of conception would likely have changed and, therefore, a different sperm and egg would have fused.

This clarification about how PNT and MST work is important because it indicates, specifically, how MRTs *prevent* mtDNA diseases, and because it shows that de Melo-Martin is incorrect when she assumes that MRTs would not effectively alleviate the burdens of mtDNA diseases. When de Melo-Martin speaks about the reduction of burdens of mitochondrial disorders, she states that other means (such as basic and clinical studies on the causes, prevention, and treatment of these diseases) would be “more effective than research on MRTs.”^27^ However, if MRTs work as expected, then PNT and MST with preselected gametes would *in fact* be effective in treating mtDNA diseases, if “effective” means the successful elimination of a condition. It is difficult to see how techniques that will successfully treat mtDNA diseases can be labelled as *ineffective*.

At this point, de Melo-Martin may argue that she is not talking about how effective MRTs are, or could be, as clinical procedures, but rather how *cost-effective* they are, or could be, when compared with the cost-effectiveness of other treatments for mtDNA diseases, or for mitochondrial diseases in general. If this is true, then she is advancing an empirical claim that needs to be supported by empirical data, which she does not provide. Even if these data were available, de Melo-Martin would still need to present a compelling argument to show that we as a society have a moral duty to allocate the most resources to the most cost-effective research/treatment. And even if she presented such an argument, the medical community would need to compare *all* medical research/treatments (assuming that this cost-effectiveness rationale is restricted to medical practice) to find out which would be the most cost effective. This means that *it is not completely certain at this point* that research/treatment on MRTs would be halted under a cost-effectiveness paradigm.

The problem with the cost-effectiveness argument is that it artificially forces one to *only* compare the cost-effectiveness of research into MRTs with the cost-effectiveness of *other possible treatments* for mitochondrial diseases. To reach a conclusion, one needs to compare *all possible* medical research/treatments against one another and then see how research into MRTs fares. If this is what de Melo-Martin is arguing, then it has to be accepted that her case is at best inconclusive.

## Reasons for Disclosure

A central topic of both Harris’s and de Melo-Martin’s work is whether MRT-conceived children have a right to know their genetic origins. It is important to note that whereas Harris states that “[a] problem is often raised about whether or not resulting children have a right or a need to know *the identity of the mitochondria donor* [emphasis added]”^28^, de Melo-Martin focuses on “the alleged right to know *one’s genetic origins*? …Furthermore, I believe that talk of a right to know one’s genetic parentage imbues genetic information with very special significance and thereby contributes to promoting problematic beliefs about genetic essentialism.”^29^ This distinction is important because there is a subtle difference between the right to know the identity of the mitochondrial donor and the right to know one’s genetic origins. The latter alleged right does not seem to *necessarily imply* a right to know the *identity* of those genetic origins.

Harris rejects the idea that there is such a right, which he labels as dangerous nonsense. He contends that if everybody had the right to know who their progenitors were we would need “universal paternity testing, with all the mischief that this would entail.”^30^ He anticipates “mischief” because of the phenomenon known as “non-paternity.” Non-paternity is a concept used to describe cases in which the biological father of a child is not who it is presumed to be. This belief can be held by the child, the presumed genetic father, or the mother. According to Harris, non-paternity cases should not be a cause for concern, and he even doubts the wisdom of correcting this state of affairs. He concludes that “[m]ore mischief and anxiety would certainly be caused by recognizing a right to know, or indeed a duty to disclose, all contributors to a given genome.”^31^

De Melo-Martin is also unpersuaded by the supposed “right to know” when considering the clinical application of MRTs; however, as she states, not for the reason that Harris presents: “my disinclination to make much of this alleged right to know one’s genetic origins has nothing to do with the phenomenon of non-paternity, but simply with the fact that no compelling grounds exist to support this presumed right.”^32^

Even if we accept, *for the sake of argument*, that there is no such thing as a “right to know”^33^ one’s genetic origins or the identity of the mitochondrial donor, as de Melo-Martin and Harris maintain, it must be noted that *there are strong reasons to disclose* to someone that that person was MRT conceived. It is important to emphasize that to disclose to someone that he or she was MRT conceived is not the same as revealing who the “mitochondria donor” is, but rather it is similar to disclosing to someone that he or she was conceived with a donated gamete without revealing the donor’s identity. It is also important to point out that “to disclose to” someone should not be understood negatively, as in “*only if* X asks about the way in which she was conceived we will tell her that she was MRT conceived.” Here disclosure should be understood in a positive way: we have to “go and tell” X, at some point, that she was MRT conceived.^34^

Ravelingien and Pennings, when discussing the issues surrounding the purported “right to know” one’s genetic parents (and referencing Vardit Ravitsky’s^35^ work on the topic), touch on some of the reasons for disclosing to someone his or her genetic background: “Awareness of one’s genetic background is deemed necessary for a better understanding of and decision making about one’s health risks…Access to a full picture of one’s genetic background is also regarded as essential in terms of one’s psychological well-being and family relationships.”^36^

At this point, I will set aside issues surrounding how knowing that one was MRT conceived affects one’s psychological well-being and family relationships.^37^ Instead, I will unpack the medical reasons parents have for disclosing to their children that they were MRT conceived. If parents disclose the conditions of their conception, these children, when they are old enough, will have a better understanding of the health risks associated with their conception, or in this case of the uncertainties about such health risks.

Knowing one’s *genetic background* can be *instrumentally good*: it provides information about oneself that might otherwise not be obvious, and this information can be helpful for better assessing and managing health risks. Knowing that both my parents are recessive carriers of a Mendelian-inherited-type genetic condition provides me with information with which I can make better informed decisions regarding my lifestyle choices; that is, decisions that could benefit my health in the long term such as refraining from smoking. Although whether I have a *right* to such information is outside the scope of this article, it is true that possessing such information is instrumentally good for me and that my parents have at least *pro tanto*^38^ reasons to reveal it. This being the case, I can also affirm that *possessing the information that I was MRT conceived can be instrumentally good for me*, and, therefore, that my parents also have *pro tanto* reasons to reveal it. Furthermore, these reasons are *stronger* because the way in which I was conceived is novel and, therefore, could carry more health risks. It must be noted at this point that the advent and increasing popularity of personal genomic services (for example, 23andMe) could make it possible for people to come to know that they were MRT conceived without their parents having to disclose this to them.

Individuals who know about their MRT conception could use this information to take better care of their health, and to enable their medical team to make better decisions when investigating the causes of an illness, for example. As John Appleby says: “Disclosure is important for at least two reasons: (1) the MRT-conceived person’s own medical welfare; and (2) knowledge of having been MRT-conceived enables persons to report any medical problems back to clinicians and researchers for the sake of the wellbeing of future generations who might be conceived via MRTs.”^39^

He further provides an additional reason for disclosure: “it would save some children from the stress and anxiety of worrying about having from the same mtDNA disorders as their mothers.^40^

Harris and de Melo-Martin would agree, as would any other reasonable person, that regardless of the existence of a right to know one’s genetic origins or the identity of the mitochondrial donor, there are strong *pro tanto* reasons for disclosing to someone that that person was MRT conceived. At this point we have to conclude that, although their discussion of the supposed “right to know” is relevant and regardless of whether they are correct or not, it is important to go beyond it and take into consideration other reasons for disclosure, as just presented.

## Conclusion

De Melo-Martin and Harris have had a lively debate (which fits within the broader scope of recent work on the ethics of MRTs that deals with issues of identity,^41^ transgenerational health risks,^42^ the disclosure of MRT conception,^43^ genealogical ancestry,^44^ first in-human use,^45^ the possible use of nonhuman oocytes for PNT,^46^ and the anonymity status of the “mitochondrial donor”^47^) regarding the morality of MRT research and clinical practice. In this article I have broadened the scope of the discussion regarding mitochondrial diseases and MRTs. I showed that de Melo-Martin’s effectiveness argument is at best inconclusive.^48^ I also showed that it is methodologically important to differentiate between mitochondrial diseases in order to understand which diseases MRTs could prevent, and how MRTs would do this.

Further, I also showed that the clinical practice of PNT and MST without preselected gametes prevents mtDNA disease by creating people without an mtDNA disease, rather than by curing those who already have an mtDNA disease. PNT and MST with preselected gametes, on the other hand, can be said to cure mtDNA disease insofar as the numerical identity of the individual who will be “brought” into existence is not altered. This is relevant in relation to de Melo-Martin’s claim that other research avenues might be more “effective” in treating mtDNA diseases, despite the fact that if PNT and MST with preselected gametes were successful they would for certain be effective in treating mtDNA diseases.

Finally, I have shown that parents have strong *pro tanto* reasons for disclosing to their children that they were MRT conceived. If they do so, their children will be able to make better decisions regarding their medical welfare (this issue is heightened by the fact that at least the first generation of MRT-conceived children would be born from an experimental technique), and medical teams will be better equipped to treat them. This shows that even if there is no such thing as a “right to know,” as Harris and de Melo-Martin maintain, there are important reasons to reveal to someone that that person was MRT conceived.

The richer account of MRTs that I have presented here shows that there is much more to be said about the morality of MRT research and its clinical practice.

## Notes

1. HarrisJ. Germline manipulation and our future worlds. The American Journal of Bioethics: AJOB 2015;15(12):30–4;10.1080/15265161.2015.110416326632358 and HarrisJ. Germline modification and the burden of human existence. Cambridge Quarterly of Healthcare Ethics. 2016;25(1):6–18.2646582810.1017/S0963180115000237

2. de Melo-Martin I. When the milk of human kindness becomes a luxury (and untested) good. A reply to Harris’ unconditional embrace of mitochondrial replacement techniques. *Cambridge Quarterly of Healthcare Ethics* 2016;26(1).

3. Harris J. How to welcome new technologies: some comments on Inmaculada de Melo-Martin. *Cambridge Quarterly of Healthcare Ethics* 2016;26(1).

4. The advent of in vitro gametogenesis could change this fact, given that one day it might be possible to generate eggs from male induced pluripotent stem (iPS) cells and embryonic stem (ES) cells. See: Palacios-GonzálezC, HarrisJ, TestaG. Multiplex parenting: IVG and the generations to come. Journal of Medical Ethics 2014;40(11):752–8.2460808710.1136/medethics-2013-101810PMC4215291

5. ZevianiM, BonillaE, DeVivoDC, DiMauroS. Mitochondrial diseases. Neurologic Clinics 1989;7(1):123–56.2646519

6. Taylor RW, Taylor GA, Durham SE, Turnbull DM. The determination of complete human mitochondrial DNA sequences in single cells: implications for the study of somatic mitochondrial DNA point mutations. *Nucleic Acids Research* 2001;29(15):e74–e74.

7. See note 1, Harris 2015, at 31; Harris 2016, at 10.

8. Nuffield Council on Bioethics*. Novel Techniques for the Prevention of Mitochondrial DNA Disorders: An Ethical Review*. London: Nuffield Council on Bioethics; 2012, at vii.

9. Department of Health. *Mitochondrial Donation A Consultation on Draft Regulations to Permit the Use of New Treatment Techniques to Prevent the Transmission of a Serious Mitochondrial Disease from Mother to Child*. 2014; available at https://www.gov.uk/government/consultations/serious-mitochondrial-disease-new-techniques-to-prevent-transmission (last accessed 25 Mar 2016).

10. Appleby JB. The ethical challenges of the clinical introduction of mitochondrial replacement techniques. *Medicine Health Care Philosophy*. 2015;18(4):501–14; and see note 8, Nuffield Council on Bioethics 2012.

11. It is worth mentioning that when referring to both MST and PNT, Harris uses the term “mitochondrial replacement therapy,” whereas de Melo-Martin and I use the term “mitochondrial replacement techniques.” For a discussion on terminology see Ravitsky, V., Birko, S., & Dupras-Leduc, R. The “Three-Parent Baby”: A Case Study of How Language Frames the Ethical Debate Regarding an Emerging Technology. *The American Journal of Bioethics*. 2015;15(12):57–60.

12. See note 1, Harris 2015; Harris 2016.

13. See note 3, Harris 2016.

14. There is another technique, which I will not discuss here, that has been suggested for preventing the clinical expression of mtDNA diseases: ooplasmic transfer. It involves injecting cytoplasm from a donor’s oocyte, with healthy mitochondria, into an oocyte with diseased mitochondria. See Brenner CA, Barritt JA, Willadsen S, Cohen J. Mitochondrial DNA heteroplasmy after human ooplasmic transplantation. *Fertility and Sterility* 2000;74(3):573–8.

15. See note 2, de Melo-Martin 2016.

16. Tachibana M, Amato P, Sparman M, Woodward J, Sanchis DM, Ma H, et al. Towards germline gene therapy of inherited mitochondrial diseases. *Nature* 2013;493(7434):627–31.

17. WrigleyA, WilkinsonS, ApplebyJB. Mitochondrial replacement: ethics and identity. Bioethics. 2015;29(9):631–8.2648120410.1111/bioe.12187PMC4620703

18. Palacios-González C. Mitochondrial replacement techniques: egg donation, genealogy and eugenics. *Monash Bioethics Review*. 2016;34(1):37–51. Palacios-González, C. Ethics of Mitochondrial Replacement Techniques: A Habermasian Perspective. *Bioethics*, Forthcoming.

19. CravenL, TuppenHA, GreggainsGD, HarbottleSJ, MurphyJL, CreeLM, et al Pronuclear transfer in human embryos to prevent transmission of mitochondrial DNA disease. Nature 2010;465(7294):82–5.2039346310.1038/nature08958PMC2875160

20. See note 16, Tachibana et al. 2013.

21. See note 1, Harris 2015, at 32; Harris 2016, at 11.

22. See note 2, de Melo-Martin 2016.

23. I am not unaware of the philosophical problems surrounding early embryo individuation (i.e., fission and fusion cases). Therefore, when I talk about “curing someone,” I am not assuming that the embryo is a person, or that it cannot divide itself into two or more identical copies, or that these identical copies cannot fuse back together. Here “someone” should be understood as a shorthand for “an embryo that can give rise to a body, or bodies, that can have, or not have, an mtDNA disease.”

24. I have explored this at length elsewhere, see note 18.

25. See note 17, Wrigley et al. 2015, at 638.

26. Even when Wrigley et al. recognized the differences between PNT and MST, they failed to realize that MST with preselected gametes would also cure someone, as just explained.

27. See note 2, de Melo-Martin 2016.

28. See note 1, Harris 2016, at 12.

29. See note 2, de Melo-Martin 2016.

30. See note 1, Harris 2016, at 12.

31. See note 1, Harris 2016, at 12.

32. See note 2, de Melo-Martin 2016.

33. For a recent informative discussion about the “right to know,” see: Ravelingien A, Pennings G. The right to know your genetic parents: from open-identity gamete donation to routine paternity testing. *The American Journal of Bioethics: AJOB* 2013;13(5):33–41; and Brandt R. Why disclosure of genetic ancestry in misattributed paternity cases should be treated differently from disclosure in adoption and gamete donation. *The American Journal of Bioethics: AJOB* 2013;13(5):58–60.

34. How and when to disclose are questions beyond the scope of this article.

35. RavitskyV. Conceived and deceived: the medical interests of donor-conceived individuals. Hastings Centre Report 2012;42(1):17–22.10.1002/hast.922616395

36. See note 33, Ravelingien, Pennings 2013, at 33.

37. For a discussion of this issue see Institute of Medicine of the National Academies. *Mitochondrial Replacement Techniques: Ethical, Social, and Policy Considerations*. The National Academies Press 2016; available at http://www.nap.edu/21871 (last accessed 3 Mar 2016); see note 10, Appleby 2015; and DimondR. Techniques of donation: “three parents”, anonymity and disclosure. Journal of Medical Law and Ethics 2015;3(3):165–173.

38. “A *pro tanto* reason to φ is a reason that genuinely speaks in favour of φ-ing, but, while a *pro tanto* reason to φ favours φ-ing, it may not do so *decisively*: the overall balance of reasons may direct one to do otherwise.” Lenman J. *Reasons for Action: Justification vs. Explanation*. In: Zalta EN, ed. *The Stanford Encyclopedia of Philosophy*. Winter 2011; available at http://plato.stanford.edu/archives/win2011/entries/reasons-just-vs-expl/ (last accessed 4 Mar 2016).

39. See note 10, Appleby 2015, at 507.

40. See note 10, Appleby 2015, at 507.

41. See note 8, Nuffield Council on Bioethics 2012; note 17, Wrigley et al. 2015; note 37, Institute of Medicine of the National Academies 2016; Bredenoord AL, Dondorp W, Pennings G, De Wert G. Ethics of modifying the mitochondrial genome. *Journal of Medical Ethics* 2011;37(2):97–100; and Baylis F. The ethics of creating children with three genetic parents. *Reproductive BioMedicine Online* 2013;26(6):531–4.

42. See note 8, Nuffield Council on Bioethics 2012; note 10, Appleby 2015; note 37, Institute of Medicine of the National Academies 2016; note 41, Baylis 2013; Johnson MH. Tri-parenthood – a simply misleading term or an ethically misguided approach? *Reproductive BioMedicine Online* 2013;26(6):516–9; and Bredenoord AL, Dondorp W, Pennings G, De Wert G. Avoiding transgenerational risks of mitochondrial DNA disorders: a morally acceptable reason for sex selection? *Human Reproduction* 2010;25(6):1354–60.

43. See note 8, Nuffield Council on Bioethics 2012; note 10, Appleby 2015; and note 37, Institute of Medicine of the National Academies 2016.

44. See note 41, Baylis 2013; and note 37, Institute of Medicine of the National Academies 2016.

45. See note 8, Nuffield Council on Bioethics 2012; note 37, Institute of Medicine of the National Academies 2016; and Bredenoord AL, Braude P. Ethics of mitochondrial gene replacement: from bench to bedside. *British Medical Journal* 2010;341:c6021.

46. Baylis F. “Babies with some animal DNA in them”: a woman’s choice? *International Journal of Feminist Approaches to Bioethics* 2009;2(2):75–96.

47. Appleby JB. Should mitochondrial donation be anonymous? *Journal of Medicine and Philosophy*, Forthcoming.

48. After this paper was accepted for publication Tina Rulli’s paper ‘What Is the Value of Three-Parent IVF?’ appeared on press. I contend that the second argument that I have presented here against de Melo-Martin’s effectiveness argument could equally apply to Rulli’s cost-effectiveness argument, but I will have to leave an examination of her argument for another time. See: RulliTina. What Is the Value of Three-Parent IVF?. Hastings Center Report 2016;46(4):1–10.10.1002/hast.59427198755

